# SabR enhances nikkomycin production via regulating the transcriptional level of *sanG*, a pathway-specific regulatory gene in *Streptomyces ansochromogenes*

**DOI:** 10.1186/1471-2180-11-164

**Published:** 2011-07-20

**Authors:** Yuanyuan Pan, Linqi Wang, Xihong He, Yuqing Tian, Gang Liu, Huarong Tan

**Affiliations:** 1 The Key Laboratory of Systematic Mycology and Lichenology, Institute of Microbiology, Chinese Academy of Sciences, Beijing 100101, China; 2 State Key Laboratory of Microbial Resources, Institute of Microbiology, Chinese Academy of Sciences, Beijing 100101, China

## Abstract

**Background:**

*sabR *is a pleiotropic regulatory gene which has been shown to positively regulate the nikkomycin biosynthesis and negatively affect the sporulation of *Streptomyces ansochromogenes*. In this study, we investigate the mechanism of SabR on modulating nikkomycin production in *Streptomyces ansochromogenes*.

**Results:**

The transcription start point of *sabR *was determined by high-resolution S1 nuclease mapping and localized at the nucleotide T at position 37 bp upstream of the potential *sabR *translation start codon (GTG). Disruption of *sabR *enhanced its own transcription, but retarded the nikkomycin production. Over-expression of *sabR *enhanced nikkomycin biosynthesis in *Streptomyces ansochromogenes*. EMSA analysis showed that SabR bound to the upstream region of *sanG*, but it did not bind to the upstream region of its encoding gene (*sabR*), *sanF *and the intergenic region between *sanN *and *sanO*. DNase 1 footprinting assays showed that the SabR-binding site upstream of *sanG *was 5'-CTTTAAGTCACCTGGCTCATTCGCGTTCGCCCAGCT-3' which was designated as SARE. Deletion of SARE resulted in the delay of nikkomycin production that was similar to that of *sabR *disruption mutant.

**Conclusions:**

These results indicated that SabR modulated nikkomycin biosynthesis as an enhancer via interaction with the promoter region of *sanG*, and expanded our understanding about regulatory cascade in nikkomycin biosynthesis.

## Background

Two-thirds of all the known antibiotics are produced by *Streptomyces *which possess complex morphological differentiation [1]. Antibiotic biosynthesis is highly regulated and generally occurs in a growth-phase-dependent manner [2]. Moreover, the regulation of antibiotic biosynthesis involves complex networks that consist of pathway-specific regulatory genes, pleiotropic regulatory genes and global regulatory genes [3-5]. Over a decade of years, many transcriptional regulators have been identified and their biological functions have been revealed. Among them, the best known system under γ-butyrolactone control has been characterized in *S. griseus *[5]. Previous studies reported a model describing how A-Factor and its receptor-ArpA mediate pleiotropic effects on morphological differentiation and biosynthesis of secondary metabolites in *Streptomyces*. Binding of A-Factor to ArpA derepresses the expression of *adpA *that encodes a global transcriptional activator. AdpA initiates the expression of pathway-specific regulatory genes, such as *strR *in streptomycin biosynthesis, *griR *in grixazone biosynthesis and other genes (*sprA, sprB, sprD, sprT *[6]*and sgmA *[7]) related to aerial mycelium formation [8,9]. *Streptomyces *antibiotic regulatory proteins (SARPs) are the most common activators of antibiotic biosynthetic gene clusters. Thus, SARPs are potentially the ultimate target for some quorum-sensing signaling pathways that switch on antibiotic biosynthesis [10-16].

The peptidyl nucleoside antibiotic nikkomycin, produced by *Streptomyces ansochromogenes *7100 [17] and *Streptomyces tendae *Tü 901 [18], is a promising antibiotic against phytopathogenic fungi and human pathogens. In recent years, considerable progress has been made in understanding nikkomycin biosynthesis [13,17-21]. The *san *gene cluster for the nikkomycin biosynthesis includes over 20 open reading frames (ORFs) consisting of three deduced transcriptional units (*sanO-V*, *sanN-I *and *sanF-X*) and a pathway-specific regulatory gene (*sanG*). Among them, the role of *sanG *has been studied in *S. ansochromogenes *[13,22]. The previous work proved that *sanG *regulated nikkomycin production by controlling the transcription of the *sanO-V *and *sanN-I *operons directly, but did not control the expression of *sanF-X *operon [13]. The non-coding region of *sanG *extends to 1 kb upstream of *sanG *contains five binding sites of AdpA-L which positively controls the transcription of *sanG *[23]. Except AdpA-L, no any other factors triggering the transcriptional changes of *sanG *have been reported up to now.

A regulatory gene (*sabR*) outside of *san *cluster was cloned from *S. ansochromogenes *previously. Disruption of *sabR *retarded nikkomycin production in liquid media containing glucose or glycerol as carbon source and enhanced the sporulation of *S. ansochromogenes *[24]. The deduced product of *sabR *belongs to a large family of TetR-like proteins and it is similar to γ-butyrolactone receptor which has the features with helix-turn-helix (HTH) motif located in the N-termini and butyrolactone-binding motif in the C-termini. Most proteins of this family act as repressors of secondary metabolism in *Streptomyces *[25,26]. Recently, several genes encoded this family proteins have been found to play a positive role during morphological development and secondary metabolism, such as *tarA *[27], *crpA *[28] and *spbR *[15]. In this study, the function of SabR on the regulation of *sanG *expression was studied. These results will expand the limited understanding of regulatory mechanism during nikkomycin biosynthesis.

## Results

### Disruption of *sabR *enhanced its own transcription

**Figure 1:**
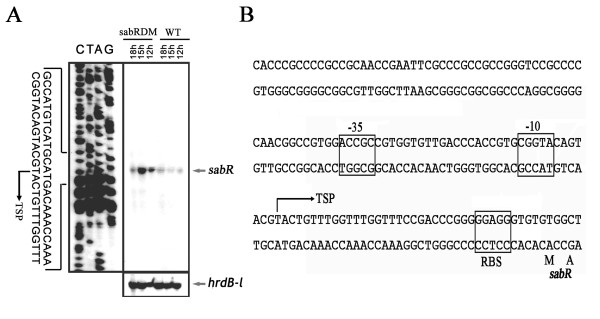
**Transcriptional analysis of *sabR***. A, High resolution S1 nuclease mapping of *sabR*. The *sabR *transcripts were detected at 12, 15 and 18 h of growth from the wild-type strain (WT) and *sabR *disruption mutant (sabRDM). The arrowhead indicates the transcription start point (TSP) of *sabR*. The *S. ansochromogenes hrdB-l *encoding a principal sigma factor was used as control. B, The nucleotide sequence of promoter region of *sabR*.

To determine the transcription start point (TSP) of *sabR *and to investigate whether *sabR *regulates its own transcription, S1 nuclease protection assay was performed. Total RNAs isolated from *S. ansochromogenes *and *sabR *disruption mutant with different time points were hybridized with 32P-labelled probe (see Methods and Table 1). The result showed that *sabR *has a single transcription start point (tsp), which is localized at the nucleotide T at position 37 bp upstream of the potential *sabR *translational start codon (GTG) (Figure 1A and 1B). Disruption of *sabR *quickly enhanced its own transcriptional level in the SP medium at 12, 15 and 18 h, whereas the transcriptional levels of *sabR *in wild-type strain tend to be weaker and constant at the same conditions (Figure 1A). After 18 h, the transcription of *sabR *in its disruption mutant was decreased to the same level as wild-type strain (data not shown). These results suggested that the expression of *sabR *could repress its own transcription at the early stage of growth.

**Table 1:** Primers used in this study

Gene and primer	Positions*	Sequence (5'-3')
***sabR***		
sab1-F	+38 to +57	CATATGGCTCAGCAGGACCGAGC
sab1-R	+697 to +681	CTCGAGGCAGGCGATGCCCGACA
sab2-F	+38 to +57	CATATGGCTCAGCAGGACCGAGC
sab2-R	+700 to +681	GGATCCTCAGCAGGCGATGCCCGACA
ER-F	-132 to -115	CCCCGTGGCACCGTTCAT
ER-R	+143 to +160	GACACCGGCCCGCTTGAG
S1R-F	-457 to -440	GCCAGAGCCGACACCACA
S1R-R	+44 to +60	ACGGCTCGGTCCTGCTG
***sanG***		
Gare1-F	-1035 to -1016	GGAATTCCGCCCGGCAGCAGCTGGACT
Gare1-R	-62 to -85	GGGGTACCAAGTGGCTCCATGTATCCGCGACC
Gare2-F	-34 to -12	GCTCTAGACCAGCTCAGGAGAATGCTCGATA
Gare2-R	+772 to +753	CCCAAGCTTCTCCGCGACCTCGTCATCAT
EG0-F	+1404 to +1423	AGGCCACCCTGCAGACGTAC
EG0-R	+1741 to +1724	GAGGAGCGTGTCGGCTTG
EG1-F	-415 to -396	GCGGGAGAACGTCACCTGTT
EG1-R	+104 to +86	CGGGTCGGCTGTGGTGAGT
EG2-F	+340 to +360	GCTCCGAGACCGTGACGAAAG
EG2-R	+763 to +743	CTCGTCATCATCAGCGTGGGT
EG3-F	+743 to +763	ACCCACGCTGATGATGACGAG
EG3-R	+1218 to +1198	GACGCGGTTTGTTGCTCTTGA
RealG-F	+1405 to +1423	GGCCACCCTGCAGACGTAC
RealG-R	+1536 to +1519	CGGGACAGGTCGAACGTG
***sanN *and *sanO***		
ENO-F	-114 to -98	TCTTGGTCGCCAGGTCC
ENO-R	+135 to +153	CTTCGGATGCTGAATGTGC
***sanF***		
EF-F	-267 to -250	CGCGCAGGTCGGCCAGGT
EF-R	+234 to +213	TACTGCTTCTCGTGCTTCGGGT
RealF-F	+70 to +87	GGTGCTGACGCTCGACTC
RealF-R	+257 to +238	TGAGGTCCACGAGGTTCATC
***hrdB***		
S1H-F	-820 to -803	GGGTACGCCCCGTCAGTG
S1H-R	+241 to +224	AGCCTTTCCCCGCTCAAT
RealH-F	+290 to +308	ACTGAGTGGCCGGAATCTG
RealH-R	+225 to +206	GTCTCTGTCATGGCGCTCAT

* The nucleotide positions for *sanG*, *sanN*, *sanO*, *sanF*, *sabR *and *hrdB *are given with respect to their transcription start points as +1. The enzyme recognition sites are underlined in the primer sequence.

### Over-expression of *sabR *accelerated nikkomycin production

**Figure 2:**
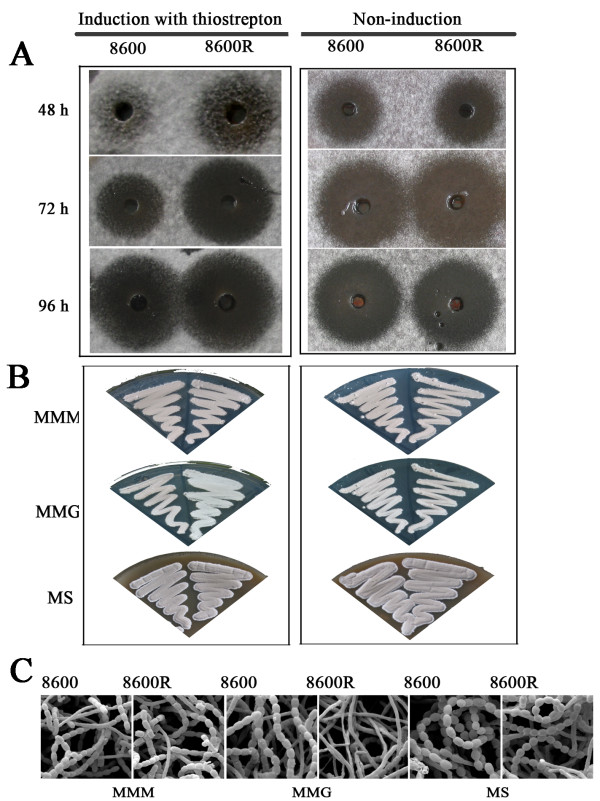
**Effects of over-expresson of *sabR *on nikkomycin biosynthesis and morphological differentiation of *S. ansochromogenes***. A, Nikkomycin bioassay of fermentation filtrates from different strains with induction of thiostrepton (the left side) or without induction of thiostrepton as control (the right side). Thiostrepton (10 μg ml^-1^) was added to the cultures after incubation for 12 h in SP medium. B, Phenotype of the *sabR *overexpressed strain (8600R) with induction of thiostrepton (the left side) or without induction of thiostrepton as control (the right side). Thiostrepton (10 μg ml^-1^) was added to the medium. C, Scanning electron micrographs of 8600R and 8600 which were grown at 28°C for 96 h in different media. MMM, MMG and MS media supplemented with thiostrepton (10 μg ml^-1^) were used. 8600, the wild-type strain carrying pIJ8600. MMM, minimal medium (MM) containing mannitol (0.5 %, w/v) as carbon source; MMG, MM containing glucose (1 %, w/v) as carbon source; MS, Mannitol soya flour medium.

In order to over-express *sabR*, the pIJ8600::*sabR *(pIJ8600R) in which the PtipA replaced the promoter of *sabR *was constructed, then this recombinant plasmid was introduced into the *Streptomyces ansochromogenes *(wild-type strain) by conjugal transfer as described previously [23]. The resulting transformant was designated as 8600R. The nikkomycin bioassay and phenotype showed that the over-expression of *sabR *accelerated nikkomycin production and delayed the morphological differentiation of 8600R in the presence of thiostrepton, whereas nikkomycin production and the morphological differentiation have no obvious difference comparing with the control in the absence of thiostrepton (Figure 2A, B and 2C). The delayed morphological differentiation of 8600R grown on MMG medium was observed by scanning electron microscopy after incubation for 96 h, and the difference was gradually disappeared after incubation for 120 h. It seems that glucose used as carbon source played an associated role in this regulatory process since the phenotype was not appeared in the media using mannitol as carbon source. These results further confirmed that *sabR *regulates the nikkomycin biosynthesis positively and morphological differentiation negatively under certain conditions.

### Disruption of *sabR *decreased the transcription of *sanG *and *sanF*

**Figure 3:**
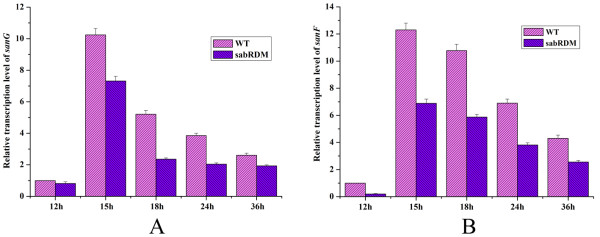
**Transcriptional analysis of *sanG *(A) and *sanF *(B) by real-time RT-PCR**. The *sanG *and *sabF *transcriptional levels were detected after fermentation for 12, 15, 18, 24 and 36 h in wild-type strain (WT) and *sabR *disruption mutant (sabRDM). Error bars were calculated from three independent samples in each reaction.

In order to know how SabR regulates nikkomycin biosynthesis in *S. ansochromogenes*, the effect of *sabR *on the transcriptions of *sanG *and *sanF-X *operon was measured by real-time quantitative PCR. The transcripts of *sanG *and *sanF *were lower in the *sabR *disruption mutant in comparison with that in the wild-type strain after fermentation for 12 h to 36 h (Figure 3). Especially, the transcripts of *sanG *and *sanF *were almost reduced to 50% in the *sabR *disruption mutant (sabRDM) in contrast to wild-type strain (WT) at 18 h. After 36 h, the transcripts of *sanG *and *sanF *in sabRDM gradually restored to the same level of WT (data not shown), suggesting that *sabR *could positively regulate the nikkomycin biosynthesis by modulating the transcription of *sanG *and *sanF *at the early stage of cell growth.

### SabR bound to the upstream region of *sanG*

**Figure 4:**
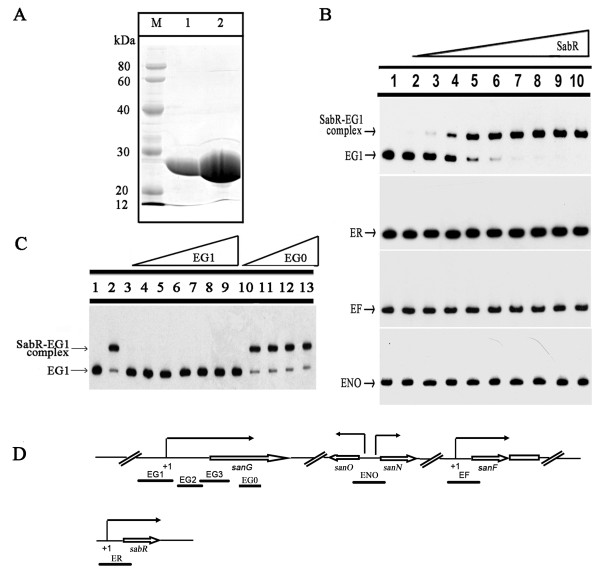
**EMSA analysis of SabR binding to the upstream of *sanG*, *sabR*, *sanN*, *sanO *and *sanF***. A, Purification of the SabR-His_6 _from *E. coli*. M, protein marker; 1 and 2, purified SabR-His_6 _protein. B, The upstream region of *sanG*, *sabR*, *sanN*, *sanO *or *sanF *was incubated with or without increasing amounts of SabR-His_6 _(lanes 1-10 contain 0, 52, 104, 130, 208, 260, 390, 520, 650 and 780 nM, respectively). C, Competition assays using unlabeled specific DNA EG1 and nonspecific competitor DNA EG0. Lanes 3-9, EMSA of 208 nM SabR-His_6 _with labeled probe and unlabeled specific competitor EG1. Lanes 10-13, EMSA of 208 nM SabR-His_6 _with labeled probe and nonspecific competitor EG0. The arrows indicate the free probe and SabR -DNA complexes. D, The gene organization of *sanG*, *sanNO*, *sanF *and *sabR*.

To determine the role of SabR in the regulation of nikkomycin biosynthesis, a series of EMSAs were performed. SabR was over-expressed in *E. coli *as His_6_-tagged protein and purified to near homogeneity by a single chromatography on Ni-NTA resin (Figure 4A). The *sanG *probes (EG1, EG2 and EG3), *sabR *probe ER, *sanF *probe EF, as well as one probe ENO covering the transcription start points of *sanN *and *sanO *were used (Figure 4D). EMSAs showed that the purified His_6_-tagged SabR bound to the probe EG1 of *sanG *to form a complex, but no complex was formed to the probe EG2 and EG3 of *sanG*. Meanwhile, no significant shift was found for probes *sabR*, *sanF*, *sanN *and *sanO*, suggesting that SabR regulated the transcription of *sabR *and *sanF *indirectly (Figure 4B). EMSAs with unlabelled specific and non-specific competitor DNA were used as controls (Figure 4C). These results indicated that SabR regulated nikkomycin biosynthesis directly by interaction with the upstream region of *sanG*. As SanG controls the transcription of *sanN *and *sanO*, SabR regulates the transcription of *sanN *and *sanO *via directly modulating the transcription of *sanG*.

### Detection of the SabR-binding sites

**Figure 5:**
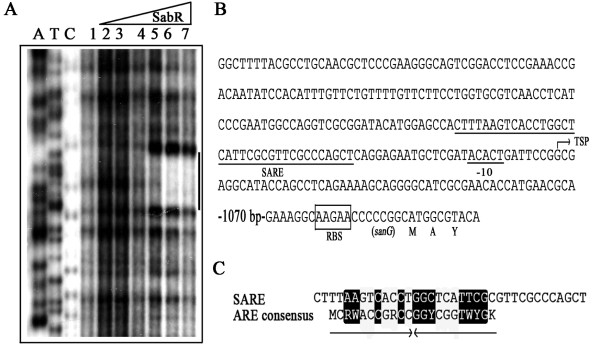
**DNase 1 footprinting analysis of SabR binding to the upstream of *sanG***. A, DNase 1 footprinting experiments. The amounts of SabR-His_6 _used in lane 1 to 7 were 0, 208, 260, 390, 520, 650 and 780 nM, respectively. The region protected against DNase 1 digestion by SabR was indicated by solid line. B, Nucleotide sequence of *sanG *promoter and SabR-binding sites. The transcription start point (TSP) of *sanG *is indicated by an arrow. The nucleotide sequence of SARE protected against DNase 1 digestion by SabR is underlined. C, Comparison of SARE with the ARE consensus sequence recognized by the *Streptomyces *γ-butyrolactone receptors. Identical residues are highlighted in black. Arrows indicate the position of the 22 bp inverted repeat sequence identified as a consensus sequence (ARE box) recognized by the γ-butyrolactone autoregulator receptor protein ArpA[39].

To identify the specific binding sites of SabR in the upstream region of *sanG*, DNase 1 footprinting assay was carried out using [γ-^32^P]-labeled probe. One region at positions -64 to -29 nucleotides was protected by SabR from DNase 1 digestion, its sequence was 5'-CTTTAAGTCACCTGGCTCATTCGCGTTCGCCCAGCT-3' (Figure 5A and 5B). This sequence showed resemblance to the reported ARE which were bound by γ-butyrolactone receptors described previously (Figure 5C), and it was designated as SARE. These results confirmed that SabR regulated nikkomycin biosynthesis by interaction with SARE sequences upstream of *sanG *directly.

### The function of SARE upstream of *sanG*

**Figure 6:**
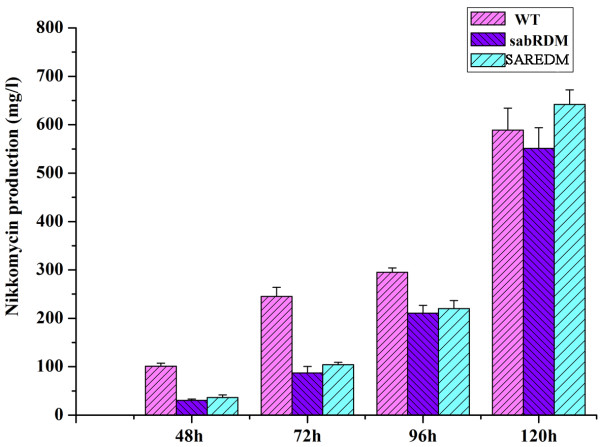
**Analysis of nikkomycin production from 48 to 120 h fermentation of the wild-type strain (WT), *sabR *disruption mutant (sabRDM) and SARE deletion strain (SAREDM)**. Error bars were calculated from three independent samples in each experiment.

In order to know the function of SARE and its relationship with SabR *in vivo*, SARE deletion mutant (SAREDM) was constructed. The bioassay showed that nikkomycin production was delayed in the SAREDM as that in the SabRDM from 48 h to 96 h fermentation. After 96 h, the nikkomycin production in SAREDM gradually restored to the level of WT, even slightly higher at 120 h (Figure 6). Therefore, SARE exhibited identical effect as *sabR *on nikkomycin production, further confirming that SabR positively regulates nikkomycin biosynthesis by interaction with SARE region upstream of *sanG *positively.

## Discussion

Our results revealed that SabR played not only the positive role for nikkomycin biosynthesis but also a negative role for morphological differentiation in *S. ansochromogenes*. Disruption of *sabR *resulted in the decrease of nikkomycin production, a phenomenon identical to pristinamycin production in *spbR *disruption mutant of *S. pristinaespiralis *[15]. However, disruption of *arpA *led to increased streptomycin biosynthesis in *S. griseus *[9] and inactivation of the *barA *led to precocious virginiamycin biosynthesis in *S. virginiae *[29]. Different γ-butyrolactone receptors have different effects on the morphological differentiation. SabR and ArpA repressed the morphological differentiation of *S. ansochromogenes *and *S. griseus *[8,24], BarA did not affect the morphological differentiation of *S. virginiae*. These results reflected that γ-butyrolactone receptors play alternative physiological roles involved in species-specific regulatory systems. In fact, two categories of homologs of autoregulator receptors are found in *Streptomyces*. One group is real receptors (ArpA, BarA, FarA and ScbR) in which binding of autoregulator is confirmed either by direct binding of natural or synthetic ligands or by gel-shift assay using crude culture filtrate [30]; the second group includes regulators (CrpA, CrpB, BarB, BarZ and so on) which show similarity to the first group receptors but lack binding of any autoregulators [31,32]. The regulators belonging to the second group widely distribute in *Streptomyces *and are usually involved in control of secondary metabolism and/or morphological differentiation. So far, no γ-butyrolactone or its analogue has been identified in *S. ansochromogenes *and no any ligands of SabR were found, but SabR could bind to the SARE region without ligand (Figure 4). The lack of SabR binding to its upstream region, in spite of the clear repression on *sabR *expression and opposite effect on nikkomycin production, implied that SabR belongs to the second group.

The demonstration that SabR interacted with the promoter region of *sanG *supported that ARE existed upstream of genes involved in antibiotic biosynthesis. The results of DNase 1 footprinting showed that SabR protected a sequence similar to those protected by PapR1, TylS and CcaR and provided the experimental evidence that γ-butyrolactone receptors recognized ARE motifs [15]. However, the disability of SabR binding to the upstream region of *sabR *was unexpected. The lack of SabR binding to its upstream region, mild effect of *sabR *disruption on *sanG *expression and almost complete recovery of nikkomycin production in *sabR *disruption mutant (sabRDM) or SAREDM at 96 h or 120 h cultivation implied that there should be one or more critical regulators other than SabR to control nikkomycin biosynthesis. Further experimental analysis will hopefully elucidate the detailed regulatory relationship between SabR and nikkomycin biosynthesis.

## Conclusions

In conclusion, this study presented detailed molecular and genetic analysis for *sabR *on the production of nikkomycin in *S. ansochromogenes*. The results revealed that the SabR regulated nikkomycin biosynthesis positively via interaction with the upstream region of *sanG*. It might be useful to expand the limited understanding of regulation exerted by SabR.

## Methods

### Strains, plasmids, media and growth conditions

The strains and plasmids used in this study are listed in Table 2. *Escherichia coli *DH5α, BL21 (DE3), ET12567 (pUZ8002), and their derivative strains were grown at 37°C in Luria-Bertani (LB) medium containing necessary antibiotics for propagating plasmids. The nikkomycin producer, *Streptomyces ansochromogenes *7100 and *sabR *disruption mutant were incubated at 28°C. For nikkomycin production, SP medium (3 % mannitol, 1 % soluble starch, 0.75 % yeast extract, and 0.5 % soy peptone, pH 6.0) was used. Liquid medium YEME and solid medium MM were prepared according to standard procedures [33]. *Alternaria longipes *was used as indicator strain for nikkomycin bioassay and incubated at 28°C in PDA medium. The plasmid pUC119::*kan*, pET23b, pIJ8600 and their derivatives were collected in our lab. *E. coli-Streptomyces *shuttle vector pKC1139 used for gene disruption was kindly provided by Prof. Keith Chater (John Innes Centre, Norwich, UK).

**Table 2:** Strains and plasmids used in this study

Strains or plasmids	relevant characteristics	Source or reference
Strains		
*S. ansochromogenes *7100	Wild-type strain	[40]
sabRDM	The *sabR *disruption mutant	[24]
*E. coli *DH5α	F- *recA *f80 d*lacZ *ΔM15	Gibco BRL
BL21(DE3)	F- *ompT hsdS gal dcm *(DE3)	Novagen
ET12567 (pUZ8002)	*recE dam dcm hsdS *Cm^r ^Str^r ^Tet^r ^Km^r^	[41]
*Alternaria longipes*	Indicator strain for nikkomycin bioassays	[40]
Plasmids		
pBluescript KS^+^	Routine cloning and subcloning vector	Stratagene
pET23b	Expression vector	Novagen
pET23b::*sabR*	*sabR *gene cloned in pET23b	This work
pIJ8600	*ori *pUC, *oriT *RK2, *int *ΦC31, *tipAp*, *tsr, apr^R^*	[33]
pIJ8600::*sabR*	*sabR *gene cloned in the induced vector of pIJ8600 which containing PtipA as promoter	This work
pKC1139	*E.coli-Streptomyces *shuttle vector	[33]
pGARE1	A 974 bp DNA fragment containing the left flank of SARE was inserted into pUC119::kan	This work
pGARE2	A 806 bp DNA fragment containing the right flank of SARE was inserted into GAREL1	This work
pGARE3	A 2.8 kb DNA fragment containing the left and right flanks of SARE and kanamycin resistance gene from pGARE2 was inserted into pKC1139	This work
pGARE4	The 1 kb kanamycin resistance gene was deleted from pGARE3	This work
pGARE5	A 1.8 kb DNA fragment containing the left and right flanks of SARE from pGARE4 was inserted into pKC1139	This work

### DNA manipulation and sequencing

Plasmids and genomic DNA were isolated from *Streptomyces *[33] or *E. coli *[34] according to the standard protocols. Intergeneric conjugation from *E. coli *ET12567 to *S. ansochromogenes *was carried out as described previously [33]. DNA sequencing was performed by Invitrogen Biotechnology Company. Database searching and sequence analysis were carried out using Artemis program (Sanger, UK), FramePlot 2.3 [35] and the program PSI-BLAST[36].

### Construction of SARE disruption mutant

Disruption of SARE was performed by gene replacement via homologous recombination. Firstly, a 974 bp DNA fragment was amplified from the genomic DNA of *S. ansochromogenes *7100 with primers Gare1-F and Gare1-R, then it was digested with *Kpn*I-*Eco*RI and inserted into the corresponding sites of pUC119::*kan *which contains the kanamycin resistance cassette to generate pGARE1. Secondly, an 806 bp DNA fragment was amplified from the genomic DNA of *S. ansochromogenes *7100 with primers Gare2-F and Gare2-R, and it was digested with *Hin*dIII-*Xba*I and inserted into the corresponding sites of pGARE1 to generate pGARE2. Thirdly, pGARE2 was digested by *Hin*dIII-*Eco*RI and the 2.8 kb DNA fragment was inserted into the corresponding sites of pKC1139 to generate a recombinant plasmid pGARE3. The plasmid pGARE3 was passed through *E. coli *ET12567 (pUZ8002) and introduced into *S. ansochromogenes *7100 by conjugation [33]. The kanamycin resistance (Kan^R^) and apramycin sensitivity (Apr^S^) colonies were selected, and the SARE disruption mutant was confirmed by PCR amplification and designated as pre-SARE. Meanwhile, the 4.9 kb DNA fragment from pGARE2 digested with *Xba*I-*Kpn*I was blunted by T4 DNA polymerase and self-ligated to generate pGARE4. Subsequently pGARE4 was digested with *Hin*dIII-*Eco*RI and inserted into the corresponding sites of pKC1139 to give pGARE5, which was then introduced into the pre-SARE strain. The kanamycin sensitive (Kan^S^) strains were selected and the SARE disruption mutants (SAREDM) were confirmed by PCR. The fidelity of all subcloned fragments was confirmed by DNA sequencing.

### Construction of a *sabR *over-expressing strain

In order to analyze the effects of over-expression of *sabR *on nikkomycin biosynthesis and morphological differentiation, a 672 bp DNA fragment containing the complete *sabR *was amplified using sab2-F and sab2-R as primers, and then it was inserted into the *Nde*I-*Bam*HI sites of pIJ8600 to generate pIJ8600::*sabR*, which was subsequently integrated into the chromosomal ΦC31 *attB *site of *S. ansochromogenes *7100 by conjugation.

### RNA isolation and S1 mapping analysis

Total RNAs were isolated from both *S. ansochromogenes *and *sabR *disruption mutant after incubation in SP medium for different times as described previously [13]. Mycelium was collected, frozen quickly in liquid nitrogen and ground into fine white powder. RNAs were then extracted using the Trizol reagent (Invitrogen, USA) according to the manufacturer's protocol. Quality and quantity of RNAs were examined by UV spectroscopy and checked by agarose gel electrophoresis. To erase the chromosomal DNA contamination, each sample was treated with DNase 1 and tested by PCR to ensure that there was no chromosomal DNA. To investigate transcription of *sabR *during nikkomycin biosynthesis, S1 protection assays were performed using the *hrdB-*like gene (*hrdB-l*) which encoded the principal sigma factor of *S. ansochromogenes *and expected to express constant during the time-course as a control. The *hrdB-l *probe was generated by PCR using the unlabeled primer S1H-F and the primer S1H-R, which was uniquely labeled at its 5' end with [γ-^32^P]-ATP by T4 polynucleotide kinase (Promega, USA). For *sabR*, the probe was generated by PCR using the radiolabeled primer S1R-R and the unlabeled primer S1R-F. The DNA sequencing ladders were generated using the *fmol *DNA cycle sequencing kit (Promega, USA) with the corresponding labeled primers. Protected DNA fragments were analyzed by electrophoresis on 6 % polyacrylamide gels containing 7 M urea.

### Real-time quantitative PCR analysis

RNA samples (1 μg) were reversedly transcribed using SuperScript™ III and random pentadecamers (N15) as described by the vendor of the enzyme (Invitrogen). Samples of cDNA were then amplified and detected with the ABI-PRISM 7000 Sequence Detection System (Applied Biosystems) using optical grade 96-well plates. Each reaction (50 μl) contained 0.1-10 ng of reversed-transcribed DNA, 25 μl Power SYBR Green PCR Master Mix (Applied Biosystems), 0.4 μM of both forward and reverse primers for *sanG *and *sanF *respectively. The PCR reactive conditions were maintained at 50°C for 2 min, 95°C for 10 min, followed by 40 cycles of 95°C for 30 s, 60°C for 1 min, fluorescence was measured at the end of each cycle. Data analysis was made by Sequence Detection Software supplied by Applied Biosystems.

### Expression and purification of SabR

The coding region of *sabR *was amplified by using primers sab1-F and sab1-R. The amplified fragment was digested with *Nde*I-*Xho*I and inserted into pET23b to generate the expression plasmid pET23b::*sabR*. After confirmed by DNA sequencing, it was introduced into *E. coli *BL21 (DE3) for protein expression. When *E. coli *BL21 (DE3) harboring pET23b::*sabR *was grown at 37°C in 100 ml LB supplemented with 100 μg ampicillin ml^-1 ^to an OD_600 _of 0.6, IPTG was added to a final concentration of 0.1 mM and the cultures were further incubated for an additional 12 h at 30°C. The cells were harvested by centrifugation at 6000 *g*, 4°C for 3 min, washed twice with binding buffer [20 mM Tris base, 500 mM NaCl, 5 mM imidazole, 5 % glycerol (pH 7.9)] and then resuspended in 10 ml of the same buffer. The cell suspension was treated by sonication on ice. After centrifugation (14000 *g *for 20 min at 4°C), the supernatant was recovered, and SabR-His_6 _was separated from the whole-cell lysate using Ni-NTA agarose chromatography (Novagen). After extensive washing with buffer [20 mM Tris base, 500 mM NaCl, 60 mM imidazole, 5 % glycerol (pH 7.9)], the SabR-His_6 _proteins were specifically eluted from the resin with 4 ml elution buffer [20 mM Tris base, 500 mM NaCl, 250 mM imidazole, 5 % glycerol (pH 7.9)] and concentrated to about 20 μg μl^-1 ^by ultrafiltration (Millipore membrane, 3 kDa cut-off size) according to the protocol provided by the manufacturer. Protein purity was determined by Coomassie brilliant blue staining after SDS-PAGE on a 12 % polyacrylamide gel. The purified protein was stored in 5 % glycerol at -70°C.

### Electrophoretic mobility-shift assays (EMSAs)

The EMSAs were performed as described previously [37]. The primers were labeled with T4 DNA polynucleotide kinase and the DNA fragments used for [γ-^32^P]-labeled probes were amplified by PCR, and then purified by using PCR purification kit (Qiagen). For EMSAs with SabR-His_6_, the *sanG *probes were generated by PCR using primers EG0-F, EG1-F, EG2-F, EG3-F and EG0-R, EG1-R, EG2-R, EG3-R, which were uniquely labeled at its 5' end with [γ-^32^P]-ATP using T4 polynucleotide kinase respectively. The *sabR*, *sanF *and *sanNO *probes were generated by PCR using unlabeled primers ER-F, EF-F, ENO-F and the radiolabeled primers ER-R, EF-R and ENO-R, respectively. During the EMSA, the [γ-^32^P]-labeled DNA probe (1000 cpm) was incubated individually with varying quantities of SabR-His_6 _at 25°C for 25 min in a buffer containing 1 μg of poly-(dI-dC) (Sigma), 20 mM Tris-base (pH 7.5), 1 mM DTT, 10 mM MgCl_2_, 0.5 μg calf BSA μl^-1 ^and 5 % glycerol in a total volume of 20 μl. After incubation, protein-DNA complex and free DNA were separated by electrophoresis on non-denaturing 4.5 % polyacrylamide gels with a running buffer containing 45 mM Tris-HCl (pH 8.0), 45 mM boric acid and 1 mM EDTA at 10 V cm^-1 ^and 4°C. Gels were dried and exposed to Biomax radiographic film (Kodak). As controls, unlabeled probe (25-fold, 50-fold, 75-fold, 100-fold, 150-fold, 175-fold and 200-fold specific competitor or 25-fold, 50-fold, 100-fold and 200-fold non-specific competitor) and labeled probe were mixed with SabR-His_6 _and incubated for 25 min at 25°C. The resulting DNA-protein complexes were then subjected to electrophoresis and autoradiography as described above. In order to quantify all probes, the probe DNA concentration was detected by ultraviolet spectrophotometer at the wavelength of 260 nm.

### DNase 1 footprinting

To characterize the SabR-binding sites upstream region of *sanG*, a DNA fragment was amplified by PCR with the labeled primer EG1-F. The footprinting reaction mixture contained 30,000 cpm of [γ-^32^P]-labeled DNA probe, 6 ng to 0.3 μg of SabR-His_6_, 2.5 μg of poly-(dI-dC) (Sigma) and 20 mM Tris-base (pH 7.5), 1 mM DTT, 10 mM MgCl_2_, 0.5 μg calf BSA μl^-1 ^and 5 % (v/v) glycerol in a total volume of 50 μl. After incubation of the mixture at 25°C for 25 min, 5.5 μl RQ1 RNase-free DNase Buffer and 0.1 U DNase 1 were added to the above reaction and the mixture was incubated for 1 min. The reaction was stopped by adding 50 μl of stop solution (20 mM EGTA, pH 8.0), and 100 μl of phenol/CH_3_Cl (1:1, v/v). After precipitation in ethanol, the pellet was washed with 75 % (v/v) ethanol and re-suspended in 5 μl of H_2_O, and then electrophoresed on a 6 % (w/v) polyacrylamide/urea gel.

### Nikkomycin bioassay

Nikkomycins produced by *S. ansochromogenes *7100 were measured by a disk agar diffusion method using *A. longipes *as indicator strain. Nikkomycins in culture filtrates were identified by HPLC analysis. For HPLC analysis, Agilent 1100 HPLC and RP C-18 were used. The detection wavelength was 290 nm. Chemical reagent, mobile phase and gradient elution process were referenced as described by Fiedler [38].

### Microscopy

The experiments of scanning electron microscopy were performed exactly as described previously [23].

## Abbreviations

EMSA: electrophoretic mobility-shift assay; SARE: autoregulatory element of *sanG*; SARP: *Streptomyces *antibiotic regulatory protein; TSP: transcription start point.

## Authors' contributions

HRT and GL conceived the project. YYP performed the experiments, LQW, XHH and YQT conducted partial data analysis. YYP, GL and HRT wrote the paper. All authors read and approved the final manuscript. The authors declare no conflict of interest.

## References

[B1] Hopwood DA Forty years of genetics with *Streptomyces*: from *in vivo *through *in vitro *to *in silico* Microbiology 1999 145 2183 2202 10517572 10.1099/00221287-145-9-2183

[B2] Chater KF *Streptomyces *inside-out: a new perspective on the bacteria that provide us with antibiotics Philos Trans R Soc Lond B Biol Sci 2006 361 761 768 10.1098/rstb.2005.1758 16627293 PMC1609407

[B3] Arias P Fernandez-Moreno MA Malpartida F Characterization of the pathway-specific positive transcriptional regulator for actinorhodin biosynthesis in *Streptomyces coelicolor *A3(2) as a DNA-binding protein J Bacteriol 1999 181 6958 6968 10559161 10.1128/jb.181.22.6958-6968.1999 PMC94170

[B4] Lee J Hwang Y Kim S Kim E Choi C Effect of a global regulatory gene, *afsR2*, from *Streptomyces lividans *on avermectin production in *Streptomyces avermitilis* J Biosci Bioeng 2000 89 606 608 10.1016/S1389-1723(00)80065-1 16232806

[B5] Horinouchi S Mining and polishing of the treasure trove in the bacterial genus *Streptomyces* Biosci Biotechnol Biochem 2007 71 283 299 10.1271/bbb.60627 17284841

[B6] Kato J Chi WJ Ohnishi Y Hong SK Horinouchi S Transcriptional control by A-factor of two trypsin genes in *Streptomyces griseus* J Bacteriol 2005 187 286 295 10.1128/JB.187.1.286-295.2005 15601713 PMC538825

[B7] Kato J Suzuki A Yamazaki H Ohnishi Y Horinouchi S Control by A-factor of a metalloendopeptidase gene involved in aerial mycelium formation in *Streptomyces griseus* J Bacteriol 2002 184 6016 6025 10.1128/JB.184.21.6016-6025.2002 12374836 PMC135398

[B8] Ohnishi Y Kameyama S Onaka H Horinouchi S The A-factor regulatory cascade leading to streptomycin biosynthesis in *Streptomyces griseus*: identification of a target gene of the A-factor receptor Mol Microbiol 1999 34 102 111 10.1046/j.1365-2958.1999.01579.x 10540289

[B9] Ohnishi Y Yamazaki H Kato JY Tomono A Horinouchi S AdpA, a central transcriptional regulator in the A-factor regulatory cascade that leads to morphological development and secondary metabolism in *Streptomyces griseus* Biosci Biotechnol Biochem 2005 69 431 439 10.1271/bbb.69.431 15784968

[B10] Wietzorrek A and Bibb M A novel family of proteins that regulates antibiotic production in Streptomycetes appears to contain an OmpR-like DNA-binding fold Mol Microbiol 1997 25 1181 1184 10.1046/j.1365-2958.1997.5421903.x 9350875

[B11] Sheldon PJ Busarow SB Hutchinson CR Mapping the DNA-binding domain and target sequences of the *Streptomyces peucetius *daunorubicin biosynthesis regulatory protein, DnrI Mol Microbiol 2002 44 449 460 10.1046/j.1365-2958.2002.02886.x 11972782

[B12] Horinouchi S AfsR as an integrator of signals that are sensed by multiple serine/threonine kinases in *Streptomyces coelicolor *A3(2) J Ind Microbiol Biotechnol 2003 30 462 467 10.1007/s10295-003-0063-z 12884127

[B13] Liu G Tian YQ Yang HH Tan HR A pathwayspecific transcriptional regulatory gene for nikkomycin biosynthesis in *Streptomyces ansochromogenes *that also influences colony development Mol Microbiol 2005 55 1855 1866 10.1111/j.1365-2958.2005.04512.x 15752205

[B14] Li R Liu G Xie ZJ He XH Chen WQ Deng ZX Tan HR PolY, a transcriptional regulator with ATPase activity, directly activates transcription of *polR *in polyoxin biosynthesis in *Streptomyces cacaoi* Mol Microbiol 2010 75 349 364 10.1111/j.1365-2958.2009.06968.x 19919670

[B15] Folcher M Gaillard H Nguyen LT Nguyen KT Lacroix P Bamas-Jacques N Rinkel M Thompson CJ Pleiotropic functions of a *Streptomyces pristinaespiralis *autoregulator receptor in development, antibiotic Biosynthesis, and expression of a superoxide dismutase J Biol Chem 2001 276 44297 44306 10.1074/jbc.M101109200 11557748

[B16] Wang LQ Tian XY Wang J Yang HH Fan KQ Xu GM Yang KQ Tan HR Autoregulation of antibiotic biosynthesis by binding of the end product to an atypical response regulator Proc Natl Acad Sci 2009 106 8617 8622 10.1073/pnas.0900592106 19423672 PMC2688989

[B17] Ling HB Wang GJ Tian YQ Liu G Tan HR SanM catalyzes the formation of 4-pyridyl-2-oxo-4-hydroxyisovalerate in nikkomycin biosynthesis by interacting with SanN Biochem Biophys Res Commun 2007 361 196 201 10.1016/j.bbrc.2007.07.016 17659257

[B18] Bruntner C Lauer B Schwarz W Möhrle V Bormann C Molecular characterization of co-transcribed genes from *Streptomyces tendae *Tü901 involved in the biosynthesis of the peptidyl moiety of the peptidyl nucleoside antibiotic nikkomycin Mol Gen Genet 1999 262 102 114 10503541 10.1007/pl00008637

[B19] Lauer B Russwurm R Schwarz W Kálmánczhelyi A Bruntner C Rosemeier A Bormann C Molecular characterization of co-transcribed genes from *Streptomyces tendae *Tü901 involved in the biosynthesis of the peptidyl moiety and assembly of the peptidyl nucleoside antibiotic nikkomycin Mol Gen Genet 2001 264 662 673 10.1007/s004380000352 11212921

[B20] Chen H Hubbard BK O'Connor SE Walsh CT Formation of beta-hydroxy histidine in the biosynthesis of nikkomycin antibiotics Chem Biol 2002 9 103 112 10.1016/S1074-5521(02)00090-X 11841943

[B21] Niu GQ Liu G Tian YQ Tan HR SanJ, an ATP-dependent picolinate-CoA ligase, catalyzes the conversion of picolinate to picolinate-CoA during nikkomycin biosynthesis in *Streptomyces ansochromogenes* Metab Eng 2006 8 183 195 10.1016/j.ymben.2005.12.002 16464627

[B22] He XH Li R Pan YY Liu G Tan HR SanG, a transcriptional activator, controls nikkomycin biosynthesis through binding to the *sanN-sanO *intergenic region in *Streptomyces ansochromogenes* Microbiology 2010 156 828 837 10.1099/mic.0.033605-0 19959580

[B23] Pan YY Liu G Yang HH Tian YQ Tan HR The pleiotropic regulator AdpA-L directly controls the pathway-specific activator of nikkomycin biosynthesis in *Streptomyces ansochromogenes* Mol Microbiol 2009 72 710 723 10.1111/j.1365-2958.2009.06681.x 19400773

[B24] Li WL Liu G Tan HR Disruption of *sabR *affects nikkomycin biosynthesis and morphogenesis in *Streptomyces ansochromogenes* Biotechnol Lett 2003 25 1491 1497 10.1023/A:1025402902098 14571971

[B25] Novakova R Kutas P Feckova L Kormanec J The role of the TetR-family transcriptional regulator Aur1R in negative regulation of the auricin gene cluster in *Streptomyces aureofaciens *CCM 3239 Microbiology 2010 156 2374 2383 10.1099/mic.0.037895-0 20466770

[B26] Hillerich B Westpheling J A new TetR family transcriptional regulator required for morphogenesis in *Streptomyces coelicolor* J Bacteriol 2008 190 1 61 67 10.1128/JB.01316-07 17965158 PMC2223726

[B27] Engel P Scharfenstein LL Dyer JM Cary JW Disruption of a gene encoding a putative γ-butyrolactone-binding protein in *Streptomyces tendae *affects nikkomycin production Appl Microbiol Biotechnol 2001 56 414 419 10.1007/s002530100621 11549012

[B28] Onaka H Nakagawa T Horinouchi S Involvement of two A-factor receptor homologues in *Streptomyces coelicolor *A3(2) in the regulation of secondary metabolism and morphogenesis Mol Microbiol 1998 28 743 753 9643542 10.1046/j.1365-2958.1998.00832.x

[B29] Nakano H Takehara E Nihira T Yamada Y Gene replacement analysis of the *Streptomyces virginiae barA *Gene encoding the butyrolactone autoregulator receptor reveals that BarA acts as a repressor in virginiamycin biosynthesis J Bacteriol 1998 180 3317 3322 9642182 10.1128/jb.180.13.3317-3322.1998 PMC107284

[B30] Takano E g-Butyrolactones Streptomyces signaling molecules regulating antibiotic production and differentiation Curr Opin Microbiol 2006 9 1 8 10.1016/j.mib.2005.12.015 16675291

[B31] Nishida H Ohnishi Y Beppu T Horinouchi S Evolution of gamma-butyrolactone synthases and receptors in *Streptomyces* Environ Microbiol 2007 9 8 1986 1994 10.1111/j.1462-2920.2007.01314.x 17635544

[B32] Xu GM Wang J Wang LQ Tian XY Yang HH Fan KQ Yang KQ Tan HR "Pseudo" gamma-butyrolactone receptors respond to antibiotic signals to coordinate antibiotic biosynthesis J Biol Chem 2010 285 35 27440 27448 10.1074/jbc.M110.143081 20562102 PMC2930742

[B33] Kieser T Bibb MJ Buttner MJ Chater KF Hopwood DA Practical Streptomyces Genetics Norwich, UK: The John lnnes Foundation 2000

[B34] Sambrook J Fritsch T Maniatis EF Molecular Cloning: A laboratory Manual Cold Spring Harbor, NY: Cold Spring Harbor Laboratory Press 1989

[B35] Ishikawa J Hotta K FramePlot: a new implementation of the frame analysis for predicting protein-coding regions in bacterial DNA with a high G+C content FEMS Microbiol Lett 1999 174 251 253 10.1111/j.1574-6968.1999.tb13576.x 10339816

[B36] Altschul SF Madden TL Schaffer AA Zhang J Zhang Z Miller W Lipman DJ Gapped BLAST and PSI-BLAST: a new generation of protein database search programs Nucleic Acids Res 1997 125 3389 3402 10.1093/nar/25.17.3389 PMC146917 9254694

[B37] Yang HH Wang LQ Xie ZJ Tian YQ Liu G Tan HR The tyrosine degradation gene *hppD *is transcriptionally activated by HpdA and repressed by HpdR in *Streptomyces coelicolor*, while *hpdA *is negatively autoregulated and repressed by HpdR Mol Microbiol 2007 65 1064 1077 10.1111/j.1365-2958.2007.05848.x 17640269

[B38] Fiedler HP Screening for new microbial products by high performance liquid chromatography using a photodiode array detector J Chromatogr 1984 316 487 494 6530423 10.1016/s0021-9673(00)96176-4

[B39] Onaka H Horinouchi S DNA-binding activity of the A-factor receptor protein and its recognition DNA sequences Mol Microbiol 1997 24 991 1000 10.1046/j.1365-2958.1997.4081772.x 9220006

[B40] Zeng HM Tan HR Li JL Cloning and function of *sanQ*: a gene involved in nikkomycin biosynthesis of *Streptomyces ansochromogenes* Curr Microbiol 2002 45 175 179 10.1007/s00284-001-0115-4 12177738

[B41] Paget MSB Chamberlin L Atrih A Foster SJ Buttner MJ Evidence that the extracytoplasmic function sigma factor *σ*^E ^is required for normal cell wall structure in *Streptomyces coelicolor *A3(2) J Bacteriol 1999 181 204 211 9864331 10.1128/jb.181.1.204-211.1999 PMC103550

